# Between Reality and Representation: Exclusion of Subpopulations in Anti‐Amyloid Therapy

**DOI:** 10.1002/alz70861_108895

**Published:** 2025-12-23

**Authors:** Ana Carolina Gomes, Sephora Sabrina Candido de Almeida, Eduardo R. Zimmer, Diogo Haddad Santos, Yngrid Dieguez Ferreira, Wyllians D Borelli

**Affiliations:** ^1^ Santa Casa de Misericórdia de São Paulo, São Paulo, São Paulo Brazil; ^2^ Federal University of Rio Grande do Sul (UFRGS), Porto Alegre, RS Brazil; ^3^ McGill Centre for Studies in Aging, Montreal, QC Canada; ^4^ Universidade Federal do Rio Grande do Sul, Porto Alegre, RS Brazil

## Abstract

**Background:**

Anti‐amyloid therapy has emerged as a promising approach for Alzheimer’s disease (AD), aiming to reduce beta‐amyloid plaques, a hallmark of the disease. However, global access to these therapies remains uneven, raising concerns about equity and representation in real‐world clinical use.

**Method:**

We analyzed real‐world data from the TriNetX platform, which included patient data globally. Patients receiving anti‐amyloid therapy (lecanemab, donanemab, or aducanumab) were retrieved from the dataset from March to April/2025. Demographic and geographic characteristics were evaluated by age, sex, race, ethnicity, and region. We also examined the clinical stage at treatment initiation to assess early versus late intervention. Descriptive analyses are shown in mean ± SD.

**Result:**

A total of 900 patients ranged from 39 to 90 years (73±8 years). Women comprised 55.11% of the cohort. The racial distribution showed 90.66% were White, while Black or African American (1.55%), Asian (2.00%), and other races (5.55%) were markedly underrepresented. Regarding ethnicity, 84.11% were non‐Hispanic, 3.56% Hispanic or Latino, and 12.33% unknown. Geographically, 885 patients (98.3%) were from the United States. Notably, 86.3% (777 patients) had a diagnosis of dementia syndrome at therapy initiation.

**Conclusion:**

Patients receiving anti‐amyloid therapy are largely biased towards White race and non‐hispanic individuals. Individuals with dementia comprised the majority of individuals receiving anti‐amyloid therapy. These data reveal significant disparities in access to anti‐amyloid therapy. The low inclusion of Black and Hispanic populations compromises the generalizability of clinical outcomes and reflects broader systemic inequities. Initiating therapy predominantly at the dementia stage suggests missed opportunities for early diagnosis and may attenuate treatment benefits. Addressing these disparities through inclusive trial design, public health policy, and cost reduction is essential to ensure equitable access and maximize the impact of disease‐modifying therapies in AD.

